# All Together Now: Pancreatic β Cells Don’t Rely on a Few to Renew

**DOI:** 10.1371/journal.pbio.0050186

**Published:** 2007-06-12

**Authors:** Richard Robinson

Stem cells have gotten a lot of press lately for their prowess at cell division and differentiation, but they certainly aren’t the only cells in the body that can replace and repair tissue. In several vital organs, including the pancreas and liver, the same cells that carry out the organ’s workaday functions also replace themselves as needed for repair or growth. But do all of these multi-talented cells contribute to the regenerative process, or are there special populations within that do most of the dividing? In a new study, Kristen Brennand, Danwei Huangfu, and Doug Melton answer that question for the insulin-producing β cells of the pancreas, and show that regeneration is a duty shared equally by all.

The authors used two complementary sets of experiments to address the question. In the first set of experiments, they tagged the entire pool of β cells with a gene for green fluorescent protein (GFP) that could be turned on and off by adding or removing doxycycline to the mice. They began with a pulse of doxycycline to turn on GFP production, and then washed it out to stop production (this classic technique is called “pulse-chase”). Thus, the entire β cell pool glowed green at the start. The authors then measured how the fluorescence was diluted over time within the dividing cells. They reasoned that if only a few β cells were responsible for most of the division, the GFP tag would be retained at full strength in most cells over time. If instead, all β cells underwent division, the tag would be evenly diluted among all progeny as division proceeded.

Even dilution is what they found, suggesting that all cells were dividing. However, several alternative explanations had to be ruled out in order to justify this conclusion. Loss of the GFP signal could occur without dilution if GFP was unstable over time, but the authors demonstrated that in (nondividing) photoreceptor cells, GFP remained intact at least six months after it was formed. They also showed that in tissues known to harbor cells dividing at highly different rates, the pulse-chase technique could distinguish the slow from the fast dividers. Finally, by observing individual β cells before and after division, the authors confirmed the GFP was equally divided between the two progeny.

**Figure:**
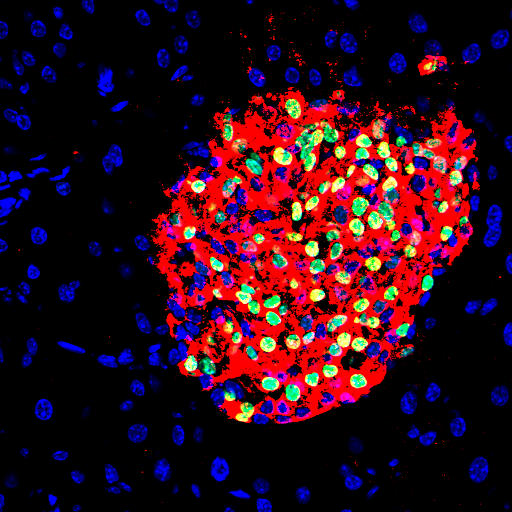
A pancreatic islet stained for insulin (red) and all nuclei (blue), including surrounding exocrine tissue. In addition, some β cell nuclei are labeled with GFP to monitor cell division.

If the first set of experiments used a “top-down” approach in its analysis of the entire β cell pool, then the second experiment examined the same question from the bottom up. Here, the authors asked whether individual β cells would give rise to few or many offspring. If a few highly replicative β cells exist among an otherwise slow-growing population, then these few should give rise to a disproportionately large number of new cells (collectively called the progenitor’s “clone”). If instead most β cells are contributing equally, then any one progenitor will only create a small clone.

To distinguish between these two outcomes, the authors left most β cells free of any marker, but tagged a small number of β cells with a different type of fluorescent label, one that was faithfully inherited and did not diminish in strength as cells divided. Thus, as the cells divided, the tagged cell’s offspring would themselves express the tag, and the increase in their number could be determined over time. The results in this experiment were consistent with those in the first experiment—there were no large clones, only many small ones.

These experiments were conducted in rodents, but are likely applicable to humans as well. Since the β cells produce insulin, and since their loss leads to type I diabetes, these results may lead to a better understanding of how the pancreas responds to this loss and how it may be manipulated to mitigate this disease process. The results may also help improve prospects for growing insulin-producing cells in culture for use in transplants to treat diabetes.

